# Biomarkers of systemic inflammation provide additional prognostic stratification in cancers of unknown primary

**DOI:** 10.1002/cam4.6988

**Published:** 2024-02-25

**Authors:** Svenja Harvey, Mark Stares, Julie‐Anne Scott, Tharun Joseph Vattam Thottiyil, Alicia‐Marie Conway, Rachel Haigh, Jackie Brown, Gillian Knowles, Sonali Dasgupta, Kai‐Keen Shiu, Claire Mitchell, Colin Barrie, Natalie Cook, Sally Clive

**Affiliations:** ^1^ University of Edinburgh, Cancer Research UK Edinburgh Centre, Institute of Genetics and Cancer, Western General Hospital Edinburgh UK; ^2^ Edinburgh Cancer Centre, NHS Lothian Western General Hospital Edinburgh UK; ^3^ Experimental Cancer Medicine Team (ECMT) The Christie NHS Foundation Trust Manchester UK; ^4^ University College London Hospitals NHS Foundation Trust London UK; ^5^ The University of Manchester, Cancer Research UK Manchester Institute Manchester UK; ^6^ The Christie NHS Foundation Trust Manchester UK; ^7^ Velindre University NHS Trust Cardiff UK

**Keywords:** cancer of unknown primary, c‐reactive protein, prognosis, Scottish inflammatory prognostic score, systemic anticancer therapy

## Abstract

**Background:**

Biomarkers of systemic inflammation have been shown to predict outcomes in patients with cancer of unknown primary (CUP). We sought to validate these findings in patients with confirmed CUP (cCUP) and explore their role alongside existing clinicopathological prognostic categories.

**Patients and Methods:**

CUP oncologist from across the United Kingdom were invited to include patients with cCUP referred to their local CUP multidisciplinary team. Patient demographics, clinical, pathological and outcome data were recorded and analysed.

**Results:**

Data were available for 548 patients from four CUP services. 23% (*n* = 124) of patients met clinicopathological criteria for favourable‐risk cCUP. On multivariate analysis c‐reactive protein (CRP) (*p* < 0.001) and the Scottish Inflammatory Prognostic Score (SIPS: combining albumin and neutrophil count) (*p* < 0.001) were independently predictive of survival. CRP and SIPS effectively stratified survival in patients with both favourable‐risk and poor‐risk cCUP based on clinicopathological features.

**Conclusions:**

Biomarkers of systemic inflammation are reliable prognostic factors in patients with cCUP, regardless of clinicopathological subgroup. We recommend that CRP or SIPS are incorporated into routine clinical assessments of patients with cCUP as a tool to aid investigation and/or treatment decision‐making across all groups. Established clinicopathological factors can then be used to inform management pathways and specific systemic anticancer therapy selection.

## 1 INTRODUCTION

Cancer of unknown primary (CUP) describes a diagnosis of metastatic disease where an established primary site cannot be identified, despite comprehensive clinical investigations.^1^, ^2^, ^3^ It is the sixth most common cancer‐related death, yet accounts only for 3–5% of cancers worldwide, reflecting its high mortality.^3^, ^4^ Patients with confirmed CUP (cCUP) have undergone comprehensive investigations, including a tumour biopsy for histopathological analysis, and review by an oncologist subspecialised in CUP.^1^, ^2^, ^5^

The selection of systemic anticancer therapy (SACT) regimens for patients with cCUP is determined by the clinicopathological features of the tumour and how closely they relate to those of a known primary.^1^, ^2^, ^3^, ^5^ Classically, cCUP is divided into two main prognostic groups: those patients with “favourable clinicopathological features” (~25%) and those with unfavourable features or “poor‐risk” cCUP. Patients with “favourable‐risk” cCUP are more likely to have chemosensitive tumours, more frequently receive SACT and have better survival (median 12.6 months).^3^, ^6^ They are often treated as their equivalent known primary tumour type within subspecialty tumour specific teams. Lower gastrointestinal, lung and renal cancer phenotypes, as well as intrahepatic cholangiocarcinoma, are now widely recognised as subgroups to be included as favourable‐risk cCUP.^2^, ^3^, ^5^, ^6^, ^7^ The majority of patients with cCUP, though, have “poor‐risk” cCUP, and empiric platinum‐doublet chemotherapy regimens remain the standard of care.^1^, ^2^, ^5^, ^7^, ^8^ Although survival is poorer in these patients (median 3.7 months), approximately 25% of those treated with SACT are alive at 1 year.^3^ Further prognostic stratification could help discussions with patients about the benefit or futility of SACT.

The role of inflammation in tumourigenesis and cancer progression is not a new concept.^9^, ^10^, ^11^ Chronic inflammation is a widely accepted hallmark of cancer with high systemic inflammatory burden consistently associated with a poorer prognosis.^9^, ^12^, ^13^ Our group has previously explored the prognostic role of several biomarkers of systemic inflammation, including c‐Reactive Protein (CRP), albumin, white cell count (WCC) neutrophil count (NC), in patients presenting with malignancy of undefined primary origin MUO or CUP to a single cancer centre. We found that the modified Glasgow Prognostic Score (mGPS; combining albumin and CRP) independently stratified outcomes in that patient population.^10^ However, CRP is not routinely tested in all new cancer patient at diagnosis, nor routinely repeated during treatments. More recently, an alternative biomarker, the Scottish Inflammatory Prognostic Score (SIPS; combing albumin and neutrophils) has been proposed.^14^, ^15^ Scores of systemic inflammation could be used as biomarkers to provide objective information regarding prognosis in patients presenting with cCUP to assist clinical management decisions. We sought to further define the prognostic significance of biomarkers of systemic inflammation in an updated cohort of patients with cCUP, including additional patients referred to specialist CUP centres across the UK.

## 2 METHODS

### 2.1 Patient population

CUP oncologists from specialist CUP centres across the UK were invited to include patients in this study. Patients referred to the Edinburgh Cancer Centre (*n* = 339), Christie NHS Foundation Trust (*n* = 161), University College Hospital (*n* = 31) and Velindre Cancer Centre (*n* = 17) CUP services between 01/09/2010 and 31/12/2021 were identified. Patients were > 18‐years and had cCUP based on published criteria relevant to the time period covered by the study^1^, ^2^

### 2.2 Prognostic biomarkers and assessments

Blood biomarkers (CRP, albumin and neutrophil count (NC)) taken within 28 days of the time of suspected cancer diagnosis, prior to any anticancer directed therapy were recorded. Cutoffs for NC (≤7.5 × 109, >7.5 × 10^9^), albumin (≥35 g/L, <35 g/L) and CRP (≤10 mg/L, >10 mg/L) calculation of mGPS (mGPS 0: albumin ≥35 g/L, CRP ≤10; mGPS 1: albumin ≥35 g/L, CRP >10; mGPS 2: albumin <35 g/L, CRP >10) and SIPS (SIPS 0: albumin ≥35 g/L, NC ≤7.5 × 10^9^; SIPS 1: albumin ≥35 g/L, NC >7.5×109; SIPS 2: albumin <35 g/L, NC >7.5 × 109) were in line with previous research^10^, ^14^

### 2.3 Statistical analysis

Overall survival was calculated from the date of suspected cancer diagnosis until death, or date of censorship (31/12/2021), if the patient was alive at this time point. Univariate analysis of survival and calculation of hazard ratios was performed using Cox proportional hazards model. Multivariate analysis of survival was carried out using a backward conditional approach: variables with a *p* > 0.10 were removed in a stepwise fashion to leave only those with an independent significant relationship with survival. The Kaplan–Meier method was used to plot survival curves and log‐rank testing was applied to assess statistically significant differences in survival.

The data was randomly split into an investigatory and validation cohort using the random‐number generator function on Excel. Analyses were carried out in the investigatory cohort and then validated in the validation cohort. The *whole* cohort was then divided according to whether patients had favourable or poor prognosis cCUP based on clinicopathological characteristics. A statistical power calculation was not carried out as all available data was used and a formal hypothesis was not being tested. Analyses were carried out in SPSS Version 25.0 (SPSS Inc).

This study uses only secondary data collected in the course of routine patient care. No patient identifiable data were used and data was anonymised for analyses. The presented work was undertaken in accordance with guidelines from the Academic and Clinical Central Office for Research and Development (ACCORD) (NHS Lothian and University of Edinburgh) and study‐specific patient consent was not required. Further, the study was conducted in accordance with the ethical principals outlined in the Declaration of Helsinki, consistent with Good Pharmacoepidemiology Practices and applicable laws and regulations of the countries where the study was conducted, as appropriate.

## 3 RESULTS

Data for 548 patients were available for analysis. The median age of the cohort was 67 (IQR 58–75) years and 51% were female (Table 1). Median survival was 5.6 (IQR 2.7–13.5) months. 42 (7.7%) patients were alive at censorship.

**TABLE 1:** Patient Characteristics.

Patient Characteristics (*n* = 548)		*n* (%)	Median (IQR)
Sex	Female	281 (51)	n/a
	Male	267 (49)	
Age (years)	≤64	218 (40)	67 (58–75)
	65–74	190 (35)	
	≥75	140 (26)	
Clinicopathological Subgroup	Favourable‐risk	124 (23)	n/a
	Poor‐risk	424 (77)	
Neutrophil count	≤7.5 × 10^9^/L	352 (64)	6.2 (4.8–9.0)
	>7.5 × 10^9^/L	196 (36)	
Albumin	≥35 g/L	322 (59)	36 (31–41)
	<35 g/L	226 (41)	
C‐Reactive protein (*n* = 355)	≤10 mg/L	73 (21)	42 (13–93)
	>10 mg/L	282 (79)	
Scottish inflammatory prognostic score	0	231 (42)	n/a
	1	212 (39)	
	2	105 (19)	
Modified glasgow prognostic score	0	73 (21)	n/a
	1	101 (28)	
	2	184 (54)	
Overall survival (all) (months)	3 months	392 (72)	5.6 (2.7–13.5)
	6 months	263 (48)	
	12 months	155 (28)	
Overall survival (favourable‐risk cCUP, *n* = 124)	3 months	104 (84)	11.2 (3.7–30.5)
	6 months	76 (73)	
	12 months	58 (47)	
Overall survival (poor‐risk cCUP, *n* = 424)	3 months	288 (70)	5.0 (2.5–11.5)

23% (*n* = 124) of patients met clinicopathological criteria for favourable‐risk cCUP (Table S1). Patients with favourable‐risk cCUP had improved survival compared to those with poor‐risk cCUP (11.2 (3.7–30.5) months versus 5.0 (2.5–11.5) months (*p* < 0.001)) (Figure S1). “CUP with a colorectal IHC (CK20+, CDX2+, CK7‐) or “molecular profile” was the most frequently observed favourable‐risk cCUP subgroup (*n* = 55 (44%)) and had similar survival to patients with poor‐risk cCUP (5.0 (3.2–13.5) months versus 5.0 (2.5–11.5) months (*p* = 0.564)) (2,16). Patients with “squamous cell carcinoma involving non‐supraclavicular lymph nodes” (*n* = 11 (9%)) or a “single metastatic deposit from an unknown primary” or “peritoneal adenocarcinomatosis of a serous papillary histological type in females” (*n* = 9 (7%)) had more favourable survival than other favourable‐risk cCUP subgroups (30.5 (13.2–62.2) months versus 5.3 (3.2–13.6) months (*p* < 0.001)).

Patients with poor‐risk cCUP were subgrouped by most likely primary site as previously described (Table S2).^3^ 42% (*n* = 178) of cases had features that could be consistent with a hepatobiliary or pancreatic cancer, and a further 31% (*n* = 132) had poorly differentiated/undifferentiated tumours. A small proportion of patients (*n* = 9 (2%)) with a suspected gynaecological primary not meeting criteria for a favourable‐risk cCUP had more favourable survival than other poor‐risk cCUP subgroups (13.3 (9.4–35.8) months versus 4.7 (2.4–11.4) months (*p* = 0.015)).

The relationship between constituent biomarkers (NC, albumin and CRP), mGPS or SIPS and survival was analysed in the investigatory and validation cohorts (Table 2). The proportion of patients with favourable‐risk cCUP in the investigatory and validation cohort was 23% and 22% respectively. CRP was not available for 193 (35%) of patients. In the investigatory cohort NC (*p* < 0.001), albumin (*p* < 0.001), CRP (*p* < 0.001), mGPS (*p* < 0.001) and SIPS (*p* < 0.001) were predictive of survival on univariate analysis. On multivariate analysis, only CRP (*p* < 0.001) and SIPS (*p* < 0.001) remained highly predictive of survival. These findings were replicated in the validation cohort (Table 2) and when the entire cohort was analysed (Table S3).

**TABLE 2:** The relationship between prognostic factors and overall survival in the investigatory and validation cohorts of patients with cCUP.

	Investigatory Cohort (n = 274)				Validation Cohort (n = 274)			
	Univariate		Multivariate		Univariate		Multivariate	
	HR (95% CI)	p	HR (95% CI)	p	HR (95% CI)	p	HR (95% CI)	p
Sex (Male, Female)	1.03 (0.80–1.32)	0.813			0.86 (0.70–1.10)	0.224		
Age (≤64, 65–74, ≥75)	1.16 (0.90–1.49)	0.243			1.02 (0.79–1.31)	0.909		
Neutrophil Count (≤7.5 × 10^9^/L, >7.5 × 10^9^/L)	1.87 (1.45–2.42)	<0.001			1.73 (1.32–2.25)	<0.001		
Albumin (≥35 g/L, <35 g/L	2.35 (1.81–3.04)	<0.001			2.54 (1.93–3.25)	<0.001		
C‐reactive protein (≤10 mg/L, >10 mg/L) *	2.96 (1.85–4.72)	<0.001	2.50 (1.55–4.03)	<0.001	2.50 (1.71–3.65)	<0.001	2.04 (1.35–3.07)	0.001
Scottish Inflammatory Prognostic Score (0, 1, 2)	1.88 (1.59–2.22)	<0.001	1.46 (1.18–1.78)	<0.001	1.85 (1.56–2.19)	<0.001	1.50 (1.07–2.10)	0.020
Modified Glasgow Prognostic Score (0, 1, 2)	1.80 (1.45–2.23)	<0.001			1.67 (1.38–2.02)	<0.001		

* Investigatory cohort n = 173, validation cohort *n* = 182.

Given that CRP was missing from 35% of patients we examined differences between the cohorts of patients with and without CRP (Table S4). There were no significant differences between the cohorts with respect to age, sex, clinicopathological risk group and NC risk score (*p* > 0.05). However, patients with a CRP measurement were more likely to have albumin <35 g/L (*p* < 0.001). Further, only 52% (*n* = 184) of patients had the same CRP and NC risk score, with 45% (*n* = 158) having a lower NC risk score (i.e., NC <7.5×10^9^ and CRP >10 mg/L) and 4% (*n* = 15) having a higher NC risk score (i.e. NC≥7.5×10^9^ and CRP >10 mg/L). A sensitivity analysis limited to patients with both NC and CRP was performed. On multivariate analyses only CRP and SIPS remained predictive of survival (*p* < 0.001 and *p* < 0.001 respectively) (Table S5).

**FIGURE 1:**
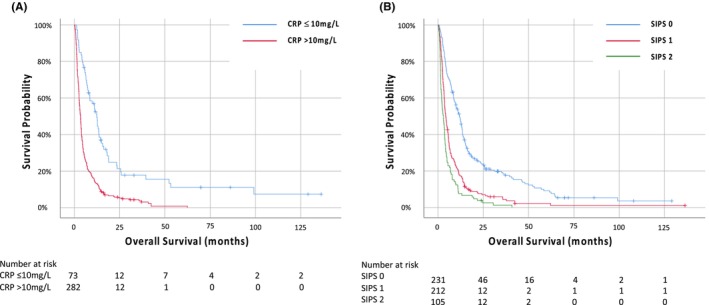
Kaplan–Meier survival curves examing the relationship between (A) CRP and (B) SIPS and overall survival in patients with cCUP.

CRP stratified survival from 3.4 (IQR 3.0–3.8) months (CRP >10 mg/L) to 12.4 (IQR 10.5–14.3) months (CRP *≤*10 mg/L) (*p* < 0.001) (Figure 1A). SIPS stratified survival from 3.0 (IQR 2.0–4.0) months (SIPS 2) to 4.3 (IQR 3.6–5.1) months (SIPS 1) to 11.8 (IQR 9.9–13.7) months (SIPS 0) (Figure 1B). For mGPS 0, 1 and 2 the median survival was 3.0, 4.0, and 12.4 months respectively (*p* < 0.001) (Figure S2).

Patients with favourable‐risk cCUP more frequently had SIPS 0 than those with poor‐risk cCUP (56% vs. 38% (*p* < 0.001)). CRP and SIPS effectively stratified survival in patients with both favourable‐risk and poor‐risk cCUP based on clinicopathological features (Table 3). Similar findings were observed with mGPS (Table S6). Notably, amongst patients with CRP < 10 mg/L there was no significant difference in survival between those with favourable‐risk versus poor‐risk cCUP (*p* = 0.136).

**TABLE 3:** The relationship between CRP or SIPS and overall survival stratified by clinicopathological‐risk subgroup in patients with cCUP.

		Favourable‐risk			Poor‐risk		
		*n* (%)	Survival (months) Median (IQR)	*p*	*n* (%)	Survival (months) Median (IQR)	*p*
SIPS (*n* = 548)	0	70 (56)	13.6 (11.4–15.7)	<0.001	161 (38)	10.0 (7.6–12.5)	<0.001
	1	37 (30)	5.3 (2.4–8.1)		175 (41)	4.2 (3.5–4.9)	
	2	17 (14)	3.9 (3.0–4.8)		88 (21)	2.3 (1.5–3.1)	
CRP (*n* = 355)	≤10 mg/L	22 (27)	12.8 (11.1–14.4)	<0.001	51 (19)	11.1(6.6–15.6)	<0.001
	>10 mg/L	59 (73)	4.1 (3.3–4.8)		223 (81)	3.0 (2.5–3.5)	

The relationship between CRP or SIPS and survival in clinicopathological favourable or poor prognostic cCUP subgroups containing >30 patients was analysed (Table 4). In each subgroup SIPS stratified survival, identifying a group with SIPS 0 who had more favourable survival than patients with SIPS 1 or 2.

**TABLE 4:** The relationship between SIPS or CRP and overall survival in clinicopathological‐risk subgroups in patients with cCUP.

	SIPS				CRP		
	0	1	2	*p*	≤10	>10	*p*
	Survival (months) Median (IQR)				Survival (months) Median (IQR)		
Favourable‐risk cCUP							
CUP with a colorectal IHC (CK20+ CDX2+ CK7‐) or molecular profile (*n* = 55)	13.1 (7.2–13.8)	3.7 (2.5–10.9)	3.6 (3.5–9.6)	*0.006*	7.4 (4.2–13.6)	3.7 (2.2–7.2)	0.061
Poor‐Risk cCUP							
Adenocarcinoma/carcinoma not otherwise specified (*n* = 60)	10.0 (6.3–21.7)	4.7 (3.3–8.6)	2.4 (1.3–6.4)	*<0.001*	n/a*	n/a*	n/a*
Poorly Differentiated/undifferentiated (*n* = 132)	11.8 (4.2–17.3)	5.0 (2.8–7.6)	1.8 (1.3–3.4)	<0.001	11.1 (2.7–23.6)	3.0 (1.6–5.5)	0.003
Squamous cell carcinoma (*n* = 31)	14.6 (7.4–31.2)	3.3 (2.2–3.8)	3.1 (1.4–6.8)	0.001	7.8 (2.5–11.3)	3.1 (2.2–3.5)	0.014
Upper Gastrointestinal/hepatobiliary or Pancreatic (*n* = 136)	6.8 (3.2–13.5)	4.0 (2.0–9.5)	2.3 (1.3–4.1)	0.003	12.8 (6.4–18.8)	2.8 (1.5–5.7)	<0.001

* Insufficient numbers for analysis.

## 4 DISCUSSION

This prospective multicentre study has confirmed that biomarkers of systemic inflammation help predict survival of patients with cCUP, validating the prognostic significance of the mGPS. Additionally, we show that simplified biomarkers of inflammation, by means of CRP alone or SIPS (a novel biomarker combining albumin and neutrophil count) provide additional useful prognostic information that may support informed clinical decision‐making in this patient group.^10^, ^14^ CRP, SIPS and mGPS are valuable biomarkers for prognostic prediction across different CUP subgroups and more distinguished than conventional clinicopathological classification in cCUP.

To our knowledge, this is the largest reported cohort of patients with well‐defined cCUP, diagnosed by specialist CUP oncologists following recognised national diagnostic guidelines. This cohort benefits from inclusion of patients at several UK regional cancer centres with different referral pathways, reflective of the diversity of CUP services throughout the country.^1^, ^3^ As our group has previously shown, survival of real‐world patients with cCUP is lower than that previously reported, reflecting the inclusion of patients who would not have been suitable for inclusion in clinical studies or for SACT.^3^, ^6^, ^8^, ^16^, ^17^, ^18^, ^19^ Standardised recording of CUP in cancer registries is an unmet need and would provide further valuable insights into real‐world outcomes across the country for this cancer.^3^, ^20^

Consistent with described cCUP populations, 23% of patients had clinicopathologically defined “favourable‐risk” cCUP.^2^, ^3^, ^5^ Median survival (11.2 months) was lower than previously reported for favourable‐risk cCUP, but was more than twice that of patients with poor‐risk cCUP in our cohort.^6^, ^16^, ^21^ However, we find that these survival differences are skewed by selected subgroups of favourable‐risk cCUP in whom median survival is 18.9–37.1 months. Indeed, in this real‐world cohort, patients with favourable‐risk poorly differentiated neuroendocrine carcinoma or colorectal‐like cCUP have similar survival outcomes to patients with poor‐risk cCUP, despite them being included in favourable‐risk classification.^2^ Notably, classical favourable‐risk clinicopathological subgroups such as “axillary node adenocarcinoma in women”, “well/moderately differentiated neuroendocrine carcinoma or “midline nodal disease in men” are minimally present in this historic real‐life cohort. These groups are now routinely referred to tumour‐group specific oncology teams (e.g., breast, neuroendocrine or germ cell) for further management. Indeed, midline disease in men and neuroendocrine carcinoma are no longer considered as CUPs in the latest European guidelines, with acknowledgment that these are likely variations of a known primary cancer type and require specialist management.^5^ Further, the latest European guidelines refine the definition of single sites of CUP to include oligometastatic disease that is amenable to local ablative therapy.^2^ This highlights the need for continual reassessment and classification of CUP as a disease entity. Our findings also support a move away from the use of broad favourable v poor‐risk cCUP descriptors which imply a survival difference towards subgroups characterised by a presumed primary site only. This consideration is likely to become more important as improved diagnostic classification, including the use of molecular profiling, identifies new treatable subgroups.

Favourable or poor‐risk clinicopathological groups are long established as the basis on which many clinical decisions are made in cCUP.^1^, ^2^, ^3^, ^5^ In particular, they inform SACT selection and entry into ongoing clinical trials, which frequently exclude patients with favourable‐risk cCUP on the basis of their superior outcomes and the ability to tailor SACT to their potential known tumour equivalent.^22^ Patients with poor‐risk cCUP, many of whom have poorly differentiated carcinoma, typically receive palliative SACT with an empirical “one‐size‐fits‐all” regime, often with limited benefit in those with poor prognosis.^3^, ^8^, ^18^ Our findings suggest that current practice, based on clinicopathological features alone, does not adequately help predict prognosis in cCUP, highlighting the need for better tools to assist clinical decision making.

The mGPS has previously been shown to predict survival in patients with MUO and its cCUP subgroup, regardless of clinicopathological prognostic group.^10^ We provide validatory evidence to support these findings. Despite not being measured in over a third of patients, we find that CRP alone is a significant independent predictor of survival in patients with cCUP. We note that 79% of patients had CRP >10 mg/L. To our knowledge we are the only group to report CRP in cCUP and advocate for external validation of this finding. CRP measurement has now been incorporated into UK guidance for minimum datasets in patients with cancer but has not yet been formally included in guidelines for assessing patients with cCUP or MUO. Although we advocate a move to do so, our data also show that SIPS predicts survival in patients with cCUP, again regardless of clinicopathological prognostic subgroup. Further, the lack of correlation between NC and CRP and the consistent finding that only CRP and SIPS were associated with survival in those patients for whom CRP were available suggests that these biomarkers are the optimal prognostic biomarkers in this patient cohort. In particular, the combination of albumin and NC as part of SIPS appears superior to the combination of albumin and CRP as part of the mGPS.

Albumin and neutrophils are established as standard investigations in cCUP and were available for all patients at each centre in this cohort.^1^ The prognostic utility of these component biomarkers of systemic inflammation is well described, as is the association between systemic inflammation and the development and progression of cancer.^9^, ^12^, ^13^ The combination of even modest reductions in albumin, reflective of chronic inflammation, combined with even modest elevations of markers of acute inflammation such as CRP and neutrophils, appear to be important predictors of survival in patients with cancer.^10^, ^12^, ^13^, ^14^, ^15^, ^23^ A key strength of these biomarkers is that they are easy to measure, readily available in routine clinical practice and simple to interpret, using well validated normal‐range cutoff values.

We find that in patients with high levels of systemic inflammation, as evidenced by CRP >10 mg/L, SIPS 2 (albumin <35 g/L and neutrophils >7.5 × 10^9^/L) or mGPS 2 (albumin <35 g/L and CRP >10 mg/L), median survival was up to only 4.1 months. In this study all biomarker measurements were recorded at the time of suspected cancer diagnosis. This short survival time includes that spent on the diagnostic pathway, which is known to be protracted in this patient group due to diagnostic uncertainty.^3^, ^7^ Conversely, we find that low levels of systemic inflammation (i.e., CRP≤10 mg/L, SIPS 0 albumin ≥35 g/L and neutrophils ≤7.5×10^9^/L, mGPS 0 albumin ≥35 g/L and CRP ≤10 mg/L) are associated with more favourable survival, regardless of clinicopathological risk group or subgroups of patients within each subgroup. Significantly, these biomarker scores highlight patients with clinicopathologically defined poor‐risk cCUP who have similar survival to those with clinicopathological favourable‐risk cCUP. Equally, these biomarkers identify patients with clinicopathological favourable‐risk cCUP who's survival is just as short as those with clinicopathologically defined poor‐risk cCUP. We agree that classifying patients into clinicopathological subgroups to ensure appropriate referral and management by tumour specific specialists is important. However, we suggest that classification of cCUP into favourable or poor prognosis is better made by assessment of biomarkers of systemic inflammation.

Performance status (PS) is the current gold standard for the assessment of prognosis, fitness for investigations, treatment and response to treatment in patients with cancer, but is a highly subjective measure.^24^, ^25^ A key strength of the biomarkers of systemic inflammation described is that they provide additional objective, readily reproducible information. Used in conjunction with existing routine clinical assessments, including PS, they may better inform earlier discussions with patients about their options. Identification of poor prognostic factors may help steer discussions away from SACT and prompt early referral to palliative care services, which has been shown to improve patient quality and quantity of life outcomes.^26^, ^27^, ^28^, ^29^ Recent reports have demonstrated that up to 90% of patients with cCUP harbour potentially targetable mutations.^30^, ^31^, ^32^, ^33^ Clinical studies, such as the CUPISCO trial (NCT03498521), assessing the efficacy of treatment with targeted‐therapies and immune‐checkpoint inhibitors, based on molecular profiling in patients with cCUP are yet to report.^22^

There is currently limited evidence to support the use of molecular profiling to identify a tissue of origin with which to guide site‐specific SACT.^34^, ^35^, ^36^, ^37^ Indeed, the latest European guidelines provide no recommendation for the use of such approaches.^5^ The use of diagnostic liquid‐biopsy techniques utilising circulating tumour DNA (ctDNA) have demonstrated their feasibility in patients with cCUP, overcoming recognised difficulties in obtaining sufficient tumour tissue for analyses in this patient group.^7^, ^22^, ^38^ With advances in this field it is prudent to suggest that such molecular analyses may enter routine clinical practice in the future. However, these investigations come with additional financial cost and as yet are not routinely available outside of clinical studies in the UK. In a resource limited healthcare system improved prognostication for patients with cCUP may help stratify those most likely to benefit from molecular profiling investigations. They may also help stratify or aid selection of patients for entry into clinical trials, where the need for stringent screening investigations often delays the initiation of SACT, with the risk of clinical decline during workup. Crucially, biomarkers of systemic inflammation, including CRP, SIPS and mGPS, predict survival in patients with cancer treated with cytotoxic chemotherapy, targeted therapy or immune‐checkpoint inhibitors.^12^, ^13^, ^14^, ^15^ In addition to lactate dehydrogenase (LDH) and alkaline phosphatase (ALP), which are traditionally included as prognostic markers in CUP, albumin and neutrophils are routinely collected in all clinical trial datasets.^39^, ^40^ We highlight the opportunity for CUPISCO to test the prognostic power of biomarkers of systemic inflammation, including LDH and SIPS, in predicting treatment outcomes in a prospectively evaluated multinational cohort of fit patients with cCUP.

Several limitations of this study are noted. Although patients from several large CUP centres are included, the majority of patients (62%) were recruited from the Edinburgh Cancer Centre. Data from a proportion of these patients were included in a subgroup of a previous study assessing the prognostic role of a more limited range of biomarkers of systemic inflammation in patients presenting with MUO.^10^ Despite this, 52% of patients investigated herein are novel. This study also benefits from its prospective design and the use of a *validation* cohort to reinforce statistically significant results of the *investigatory* cohort. Although an internal validation cohort was used, it was derived randomly from the entire population to ensure that the cohorts were balanced with respect to recruitment centres, their varying referral pathways and the proportion of favourable‐risk patients. We also highlight those patients included in this study followed the UK guidelines for the definition of CUP, including only patients with cCUP who had been formally reviewed by an oncologist with a specialist interest in CUP.^1^ Further, we note that the results of this study may not be generalizable to other races, ethnicities or countries which may have differing treatment practices and should be confirmed in other populations. Finally, we recognise that in studies of CUP patients fit enough to receive SACT, treatment of CUP is associated with better survival, but unfortunately this data was not available for all patients in this cohort so has not been investigated.

## 5 CONCLUSIONS

Our findings demonstrate the prognostic utility of biomarkers of systemic inflammation in patients with cCUP, regardless of traditionally described clinicopathological prognostic subgroup. We recommend that biomarkers (CRP, SIPS or mGPS) are incorporated into routine clinical assessments of patients with cCUP as a tool to aid investigation and/or treatment decision‐making across all groups. Established clinicopathological factors can then be used to inform management pathways and specific SACT selection.

## AUTHOR CONTRIBUTIONS

MS and SC conceived the idea. SH, JS, TT, RH, JB, SD led the primary data collection. SH and MS were responsible for data cleaning and MS analysed the data. SH, MS and SC prepared the draft manuscript. NC and AMC and provided significant intellectual input and advice in the re‐draft of the manuscript. All authors approved the final version of the manuscript.

## FUNDING INFORMATION

This work was supported by the University of Edinburgh, Cancer Research UK Edinburgh Centre, Institute of Genetics and Cancer, Edinburgh, UK.

## CONFLICT OF INTEREST STATEMENT

NC—outside the scope of the work presented, research funding/educational research grants have been received by the Experimental Cancer Medicine Team (PI: Cook) from AstraZeneca, Bayer, Pfizer, Orion, Taiho Oncology, Roche, Starpharma, Eisai, RedX, UCB, Boeringher, Merck, Stemline Tarveda and Avacta. The remaining authors have no conflicts to declare.

## ETHICS STATEMENT

The presented work was in accordance with guidelines from ACCORD (Academic and Clinical Central Office for Research and Development, NHS Lothian and University of Edinburgh) and ECC CUP‐specific consent was not required.

## Supporting information


Data S1.

## Data Availability

The datasets used and/or analysed during the current study are available from the corresponding author in reasonable requests.
