# Supplementary material for: Biomarkers of systemic inflammation provide additional prognostic stratification in cancers of unknown primary
Source: Cancer Med. 2024 Feb 25;13(3):e6988. doi: 10.1002/cam4.6988 (PMC10895198; doi:10.1002/cam4.6988)
Supplement: Supplementary file 1 — Data S1. [file CAM4-13-e6988-s001.docx]

**Supplemental Table 1:** Clinicopathological subgroup of patients with favourable-risk cCUP

| **Favourable cCUP Subgroup** | **n (%)** | **Survival (months) (median (IQR))** |
| --- | --- | --- |
| **Poorly differentiated neuroendocrine carcinoma of unknown primary** | 24 (19) | 5.5 (2.6-13.1) |
| **Peritoneal adenocarcinomatosis of a serous papillary histological type in females** | 9 (7) | 18.9 (16.4-48.0) |
| **Squamous cell carcinoma involving non-supraclavicular cervical lymph nodes** | 11 (9) | 37.1 (17.3-61.6) |
| **CUP with a colorectal IHC (CK20+ CDX2+ CK7-) or molecular profile** | 55 (44) | 5.0 (3.2-13.5) |
| **Single metastatic deposit from unknown primary** | 23 (19) | 30.5 (5.4-64.5) |
| **Men with blastic bone metastases or IHC/serum PSA expression** | 2 (2) | n/a |

**Supplemental Figure 1:** Kaplan-Meier survival curves examing the relationship between cCUP clinicopathological risk subgroup and overall survival (log-rank p<0.001).

**Supplemental Table 2:** Clinicopathological subgroup of patients with poor-risk cCUP

| **Poor Prognosis cCUP Subgroup** | **n (%)** | **Survival (months) (median (IQR))** |
| --- | --- | --- |
| **Breast/Urological** | 9 (2) | 6.3 (3.0-14.5) |
| **Adenocarcinoma/Carcinoma Not Otherwise Specified** | 60 (14) | 6.3 (3.3-11.1) |
| **Gynaecological** | 9 (2) | 13.3 (9.4-35.8) |
| **Lung/Hepatobiliary or Pancreatic** | 22 (5) | 2.7 (2.4-9.9) |
| **Lung/Upper Gastrointestinal/Hepatobiliary or Pancreatic** | 20 (5) | 3.5 (1.7-6.7) |
| **Poorly Differentiated/undifferentiated** | 132 (31) | 5.0 (2.5-12.2) |
| **Small Cell Carcinoma** | 5 (12) | 9.8 (6.7-10.0) |
| **Squamous Cell Carcinoma** | 31 (7) | 4.1 (2.5-14.7) |
| **Upper Gastrointestinal/Hepatobiliary or Pancreatic** | 136 (32) | 4.5 (2.3-11.1) |

**Supplemental Table 3:** The relationship between prognostic factors and overall survival in all patients with cCUP. *n=355

|  | **All Patients (n=548)** | | | |
| --- | --- | --- | --- | --- |
|  | **Univariate** |  | **Multivariate** |  |
|  | **HR (95% CI)** | ***p*** | **HR (95% CI)** | ***P*** |
| **Sex (Male, Female)** | 0.95 (0.80-1.13) | 0.549 |  |  |
| **Age (*≤64, 65-74,* ≥75)** | 1.07 (0.89-1.28) | 0.480 |  |  |
| **Neutrophil Count (*≤7.5x10^9^/L, >7.5x10^9^/L)*** | ***1.80 (1.49-2.30)*** | ***<0.001*** |  |  |
| **Albumin (≥35g/L, <35g/L** | ***2.42 (2.02-2.91)*** | ***<0.001*** |  |  |
| **C-reactive protein (*≤10mg/L, >10mg/L) **** | ***2.61 (1.96-3.49)*** | ***<0.001*** | ***2.15 (1.59-2.91)*** | ***<0.001*** |
| **Scottish Inflammatory Prognostic Score (0, 1, 2)** | ***1.85 (1.65-2.08)*** | ***<0.001*** | ***1.42 (1.23-1.65)*** | ***<0.001*** |
| **Modified Glasgow Prognostic Score (0, 1, 2)** | ***1.72 (1.49-1.98)*** | ***<0.001*** |  |  |

**Supplemental Table 4: Patient characteristics by CRP measurement status**

| **Patient Characteristics** | | **No CRP Measurement Available**  **n=193** | **CRP Measurement Available**  **N=355** | ***p*** |
| --- | --- | --- | --- | --- |
| **Sex** | **Female** | 102 | 179 | 0.587 |
|  | **Male** | 91 | 176 |  |
| **Age (years)** | ***≤64*** | 82 | 136 | 0.692 |
|  | ***65-74*** | 74 | 116 |  |
|  | **≥75** | 74 | 103 |  |
| **Clinicopathological Subgroup** | **Favourable-risk** | 43 | 81 | 0.886 |
|  | **Poor-risk** | 150 | 274 |  |
| **Neutrophil Count** | ***≤7.5x10^9^/L*** | 134 | 218 | 0.061 |
|  | ***>7.5x10^9^/L*** | 59 | 137 |  |
| **Albumin** | **≥35g/L** | 168 | 154 | <0.001 |
|  | **<35g/L** | 25 | 201 |  |

**Supplemental Table 5:** The relationship between prognostic factors and overall survival in the investigatory and validation cohorts of patients with cCUP for whom a CRP measurement was available

| **n=355** | **Univariate** | | **Multivariate** | |
| --- | --- | --- | --- | --- |
|  | **HR (95% CI)** | ***p*** | **HR (95% CI)** | ***p*** |
| **Sex (Male, Female)** | 1.03 (0.83-1.27) | 0.820 |  |  |
| **Age (≤*64, 65-74,* ≥75)** | 1.02 (0.90-1.16) | 0.751 |  |  |
| **Neutrophil Count (≤*7.5x10^9^/L, >7.5x10^9^/L)*** | ***1.69 (1.35-2.11)*** | ***<0.001*** |  |  |
| **Albumin (≥35g/L, <35g/L** | ***1.96 (1.56-2.45)*** | ***<0.001*** |  |  |
| **C-reactive protein (≤*10mg/L, >10mg/L)*** | ***2.67 (1.99-3.57)*** | ***<0.001*** | ***2.18 (1.60-2.93)*** | ***<0.001*** |
| **Scottish Inflammatory Prognostic Score (0, 1, 2)** | ***1.64 (1.42-1.90)*** | ***<0.001*** | ***1.44 (1.24-1.68)*** | ***<0.001*** |
| **Modified Glasgow Prognostic Score (0, 1, 2)** | ***1.73 (1.50-1.99)*** | ***<0.001*** |  |  |

**Supplemental Figure 2:** Kaplan-Meier survival curves examing the relationship between mGPS and overall survival in patients with cCUP

|  | | **Favourable-risk (n=81)** | | | **Poor-risk (n=274)** | | |
| --- | --- | --- | --- | --- | --- | --- | --- |
|  |  | **n (%)** | **Survival (months) Median (IQR)** | ***p*** | **n (%)** | **Survival (months) Median (IQR)** | ***p*** |
| **mGPS** | **0** | 22 (27) | 12.8 (6.2-52.3) | ***<0.001*** | 51 (19) | 11.1 (5.7-18.8) | ***<0.001*** |
|  | **1** | 28 (35) | 7.2 (3.4-13.5) |  | 73 (27) | 3.5 (2.4-8.6) |  |
|  | **2** | 31 (38) | 3.7 (2.2-5.5) |  | 150 (55) | 2.8 (1.5-4.9) |  |

**Supplemental Table 6:**  The relationship between mGPS and overall survival stratified by clinicopathological-risk subgroup in patients with cCUP
